# Case Report: Managing the postoperative exposure of a non-resorbable membrane surgically

**DOI:** 10.12688/f1000research.14939.1

**Published:** 2018-05-31

**Authors:** Abdullah S. Almutairi

**Affiliations:** 1 Department of Periodontology and Oral Medicine, College of Dentistry, Qassim University, Unizah, 51911, Saudi Arabia

**Keywords:** Periodontal, GBR, membrane, exposure, implant, bone.

## Abstract

Alveolar ridge deformities can be caused by several factors. Managing alveolar deformities prior to implant placement is essential to increase bone width, height or both. Several techniques and materials are now available to perform ridge augmentation procedures. The postoperative exposure of the membrane is the most frequent postoperative complications of ridge augmentation procedures. The present case describes the horizontal ridge augmentation procedure and the outcome of surgical attempt to manage post-operative membrane exposure, and shows the unpredictability of managing postoperative membrane exposure surgically.

## Introduction

Tooth replacement is now easily dealt with using implant placement, which has been a revolution in the past two decades. To place an implant into the alveolar bone, it is imperative to have a sound and stable foundation of bone. Alveolar ridge deformities are very common and may arise due to several causes, including periodontal disease, traumatic extraction, periapical lesions and implant failure ^1^.

Alveolar ridge defects can be classified for diagnosis and treatment purposes. Seibert ^2^ introduced a classification system for ridge deformities. This classified the ridge deformities into three classes according to the horizontal and vertical defect components: class I, horizontal loss of tissue with normal ridge height; class II, vertical loss of tissue with normal ridge; class III, combination horizontal and vertical loss of tissue resulting in the loss of normal height and width ^2^.

Numerous techniques and materials are currently available to manage the resorbed ridge prior to implant placement. Regarding horizontal ridge augmentation, the literature has shown that horizontal bone augmentation is highly predictable, with good resultant implant survival rates ^3^.

There are several complications related to ridge augmentation, including post-operative membrane exposure, infection, sensory disturbance, additional augmentation procedures needed, and early implant failure ^4^. One of the most frequent postoperative complications of guided regeneration therapy is the membrane exposure ^5^. The present case describes the surgical attempt and its outcome to manage the membrane exposure that had occurred 4 weeks after horizontal ridge augmentation using non-resorbable membrane with particulate allograft bone (mineralized freeze-dried bone allograft (FDBA)).

## Case report

### Presentation

A 51-year-old male was referred to the Department of Periodontics, College of Dentistry, Qassim University (Buraydah, Saudi Arabia) to extract the non-restorable tooth #45 and to evaluate the site #45 and #46 for the placement of implants.

The patient had hypercholesterolemia and was taking 20 mg Lipitor (atorvastatin) tablets once daily. Dental history revealed that his lower right first molar was extracted 11 years ago due to caries.

**Figure 1.**
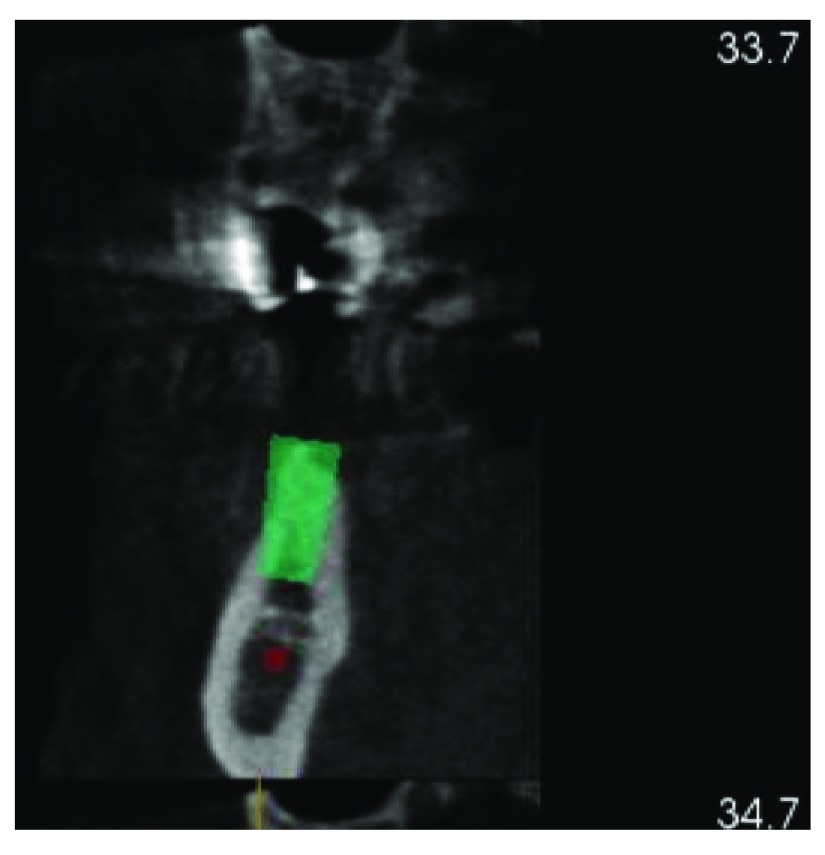
CBCT showing insufficient bone width at tooth #46.

**Figure 2.**
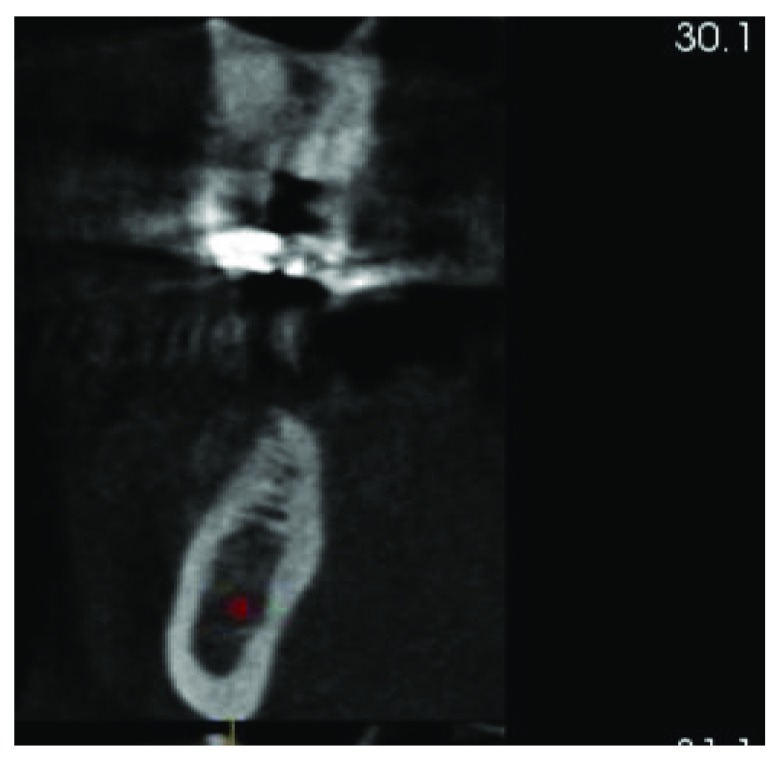
CBCT showing insufficient bone width #46.

A cone beam CT scan was taken to evaluate the ridge width and height and the location of vital structures ( Figure 1 and Figure 2). The radiographic examination revealed deformity of the ridge at site #46 (Siebert class 1). After a discussion with his referring dentist, it was decided to extract tooth #45. A free gingival graft was planned to increase the width of keratinized tissue at site #46 which was followed by ridge augmentation after waiting at least 6 weeks to allow soft tissue healing. The treatment plan was explained to the patient, and written informed consent was acquired.

### Treatment

Tooth #45 was extracted and soft tissue healing was completed about 6 weeks later.

6 weeks after the extraction of tooth #45, a free gingival graft was performed to increase the width of keratinized tissue prior to ridge augmentation.

**Figure 3.**
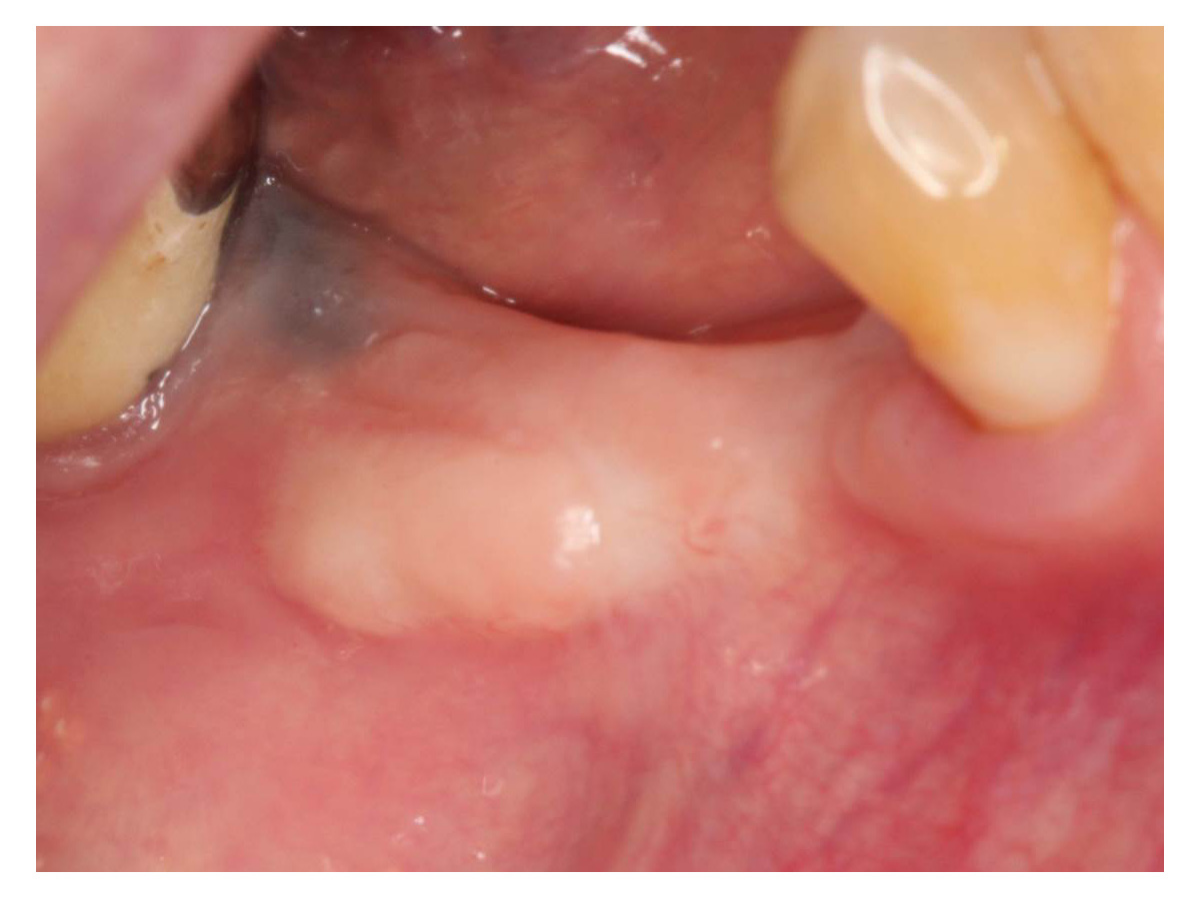
Pre- ridge augmentation.

**Figure 4.**
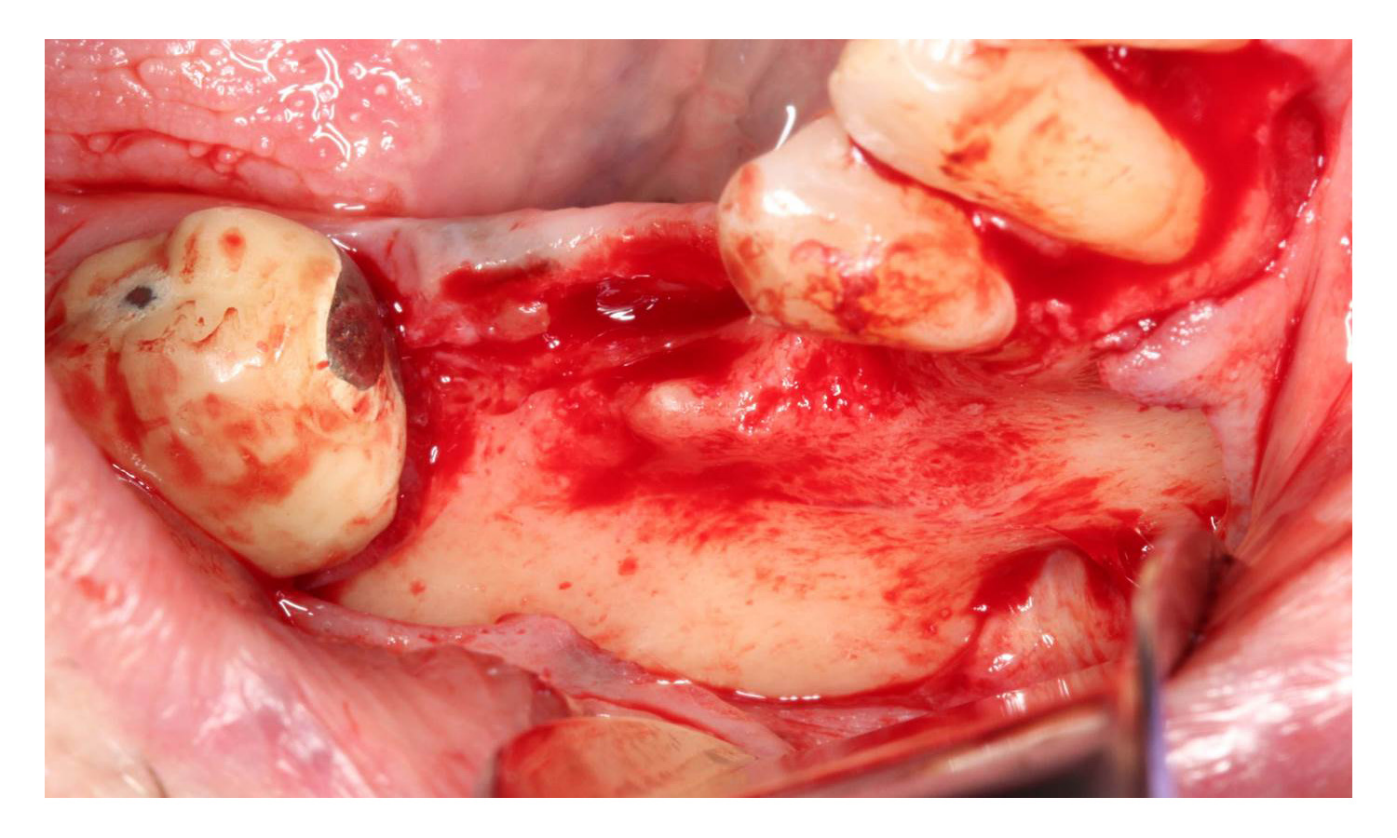
Full thickness flap reflection. The mental nerve can be seen.

At 8 weeks after the free gingival graft procedure ( Figure 3), ridge augmentation was performed using a titanium-reinforced non-resorbable polytetrafluoroethylene PTFE membrane and FDBA. Local anesthesia with 2% lidocaine and 1:100,000 epinephrine was used to anesthetize the surgical area. A full-thickness mid-crestal incision was made on the edentulous area using a sulcular extension to the distal aspect of tooth #47 and to the distal aspect of tooth #42. A vertical incision was made at the disto-buccal line angle of tooth #42. The flaps were elevated to expose the atrophic ridge ( Figure 4). Flap advancement adjacent to the mental foramen area was conducted after a full-thickness mucoperiosteal flap was elevated beyond the mucogingival junction by pushing back the flap using wet gauze until the mental nerve was located ( Figure 4). An evident horizontal bone defect was found.

**Figure 5.**
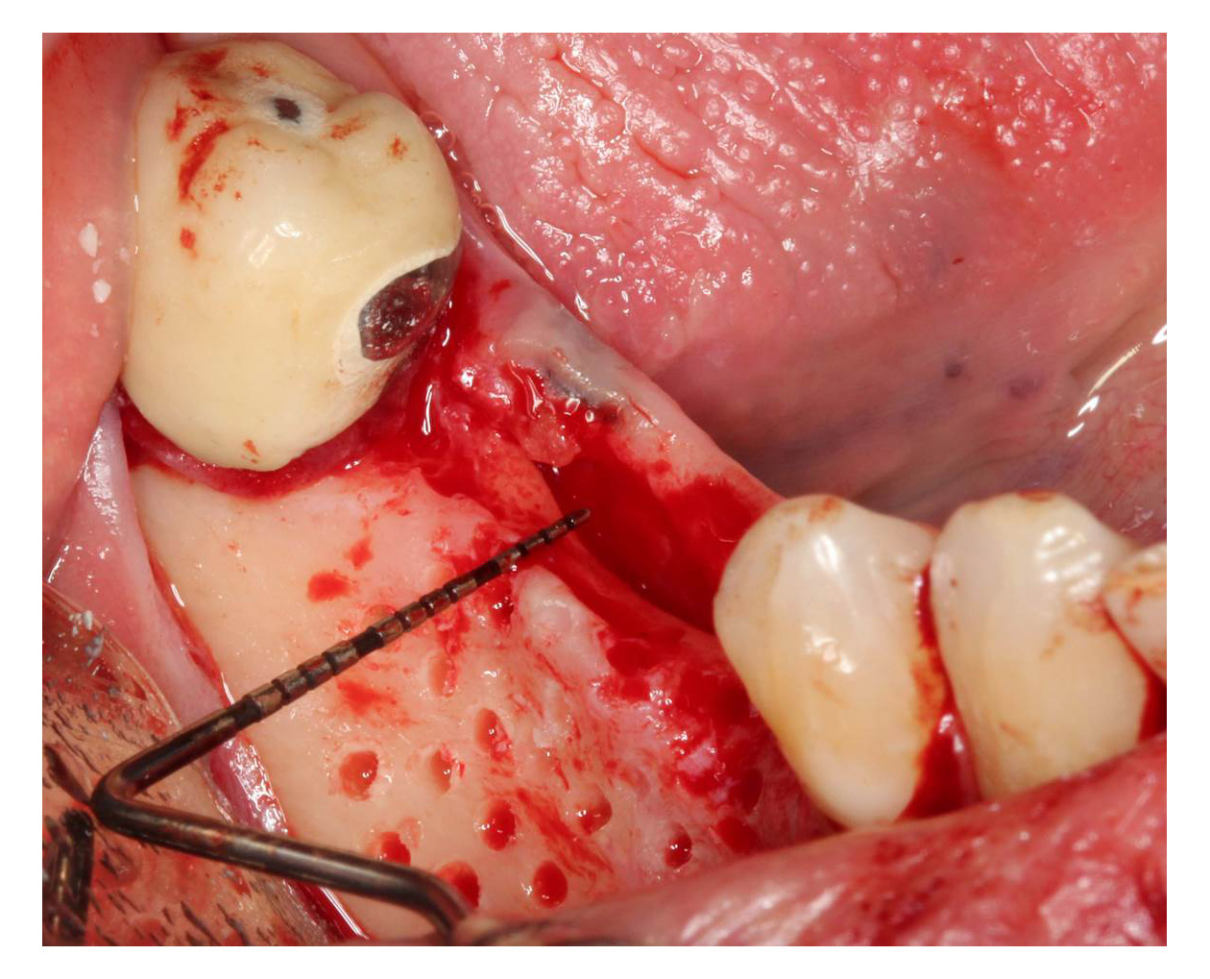
Measuring the bone width (3 mm). Decortication was performed to increase blood supply in the area.

Periosteal scoring was performed to release the buccal flap, allowing for coronal advancement of the flap. Decortication was performed in the buccal bone with a round bur ( Figure 5).

**Figure 6.**
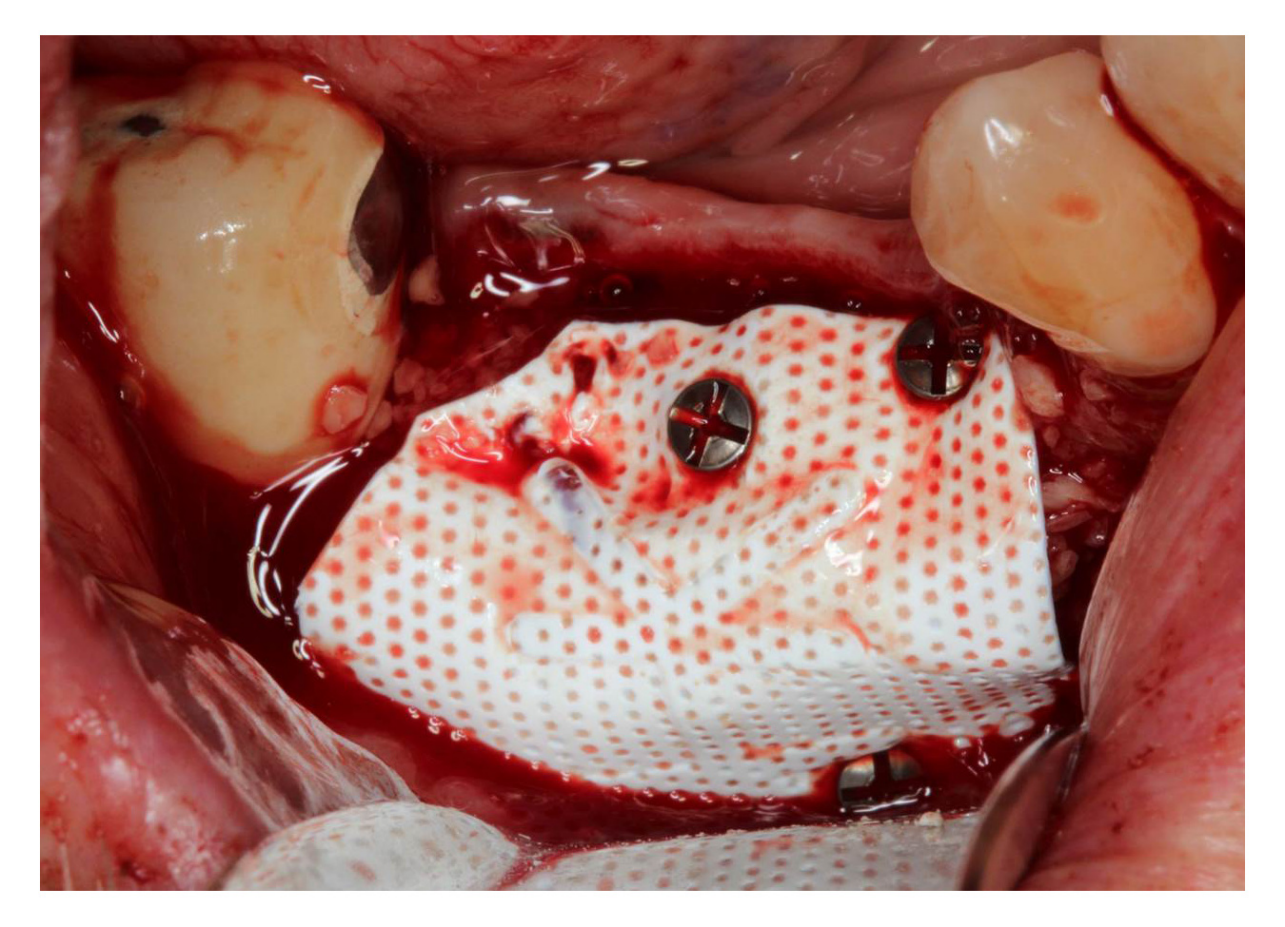
Non-resorbable membrane covering FDBA.

**Figure 7.**
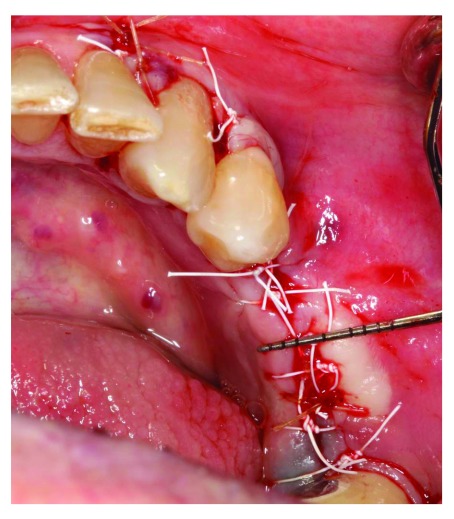
Suturing, primary closure was achieved.

A titanium-reinforced non-resorbable PTFE membrane (Cytoplast™ Barrier Membranes Ti-250) was stabilized to the buccal plate at the apical end using membrane tacks (Salvin, USA) then FDBA (OraGRAFT®, USA) was placed beneath the membrane and packed gently ( Figure 6), then the membrane coronal part was stabilized with two tacks. Flaps were sutured with 4-0 non-resorbable PTFE sutures (Cytoplast™ Sutures, Osteogenics Biomedical) ( Figure 7) Postoperative instructions (about diet, pain, bleeding and healing) and medications, including 600 mg ibuprofen three times a day for 5 days, 875 mg amoxicillin twice daily for one week and 0.12% chlorohexidine mouth wash twice a day for 2 weeks, were given to the patient.

### Follow-up and outcomes

**Figure 8.**
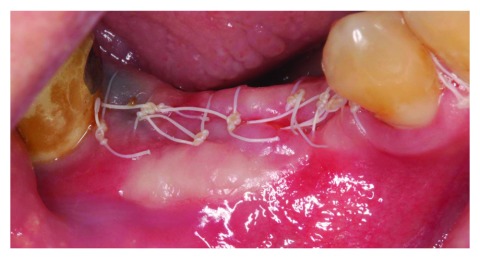
The patient at 2 weeks follow-up.

At the 2-week follow up point, the surgical site was healing well, with no sign of infection ( Figure 8).

**Figure 9.**
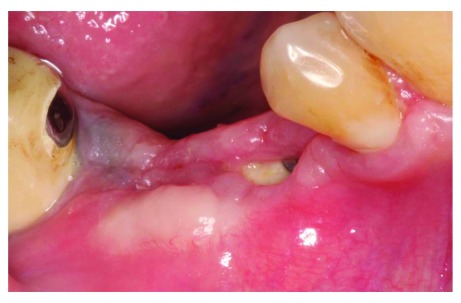
The patient at 4 weeks follow-up.

**Figure 10.**
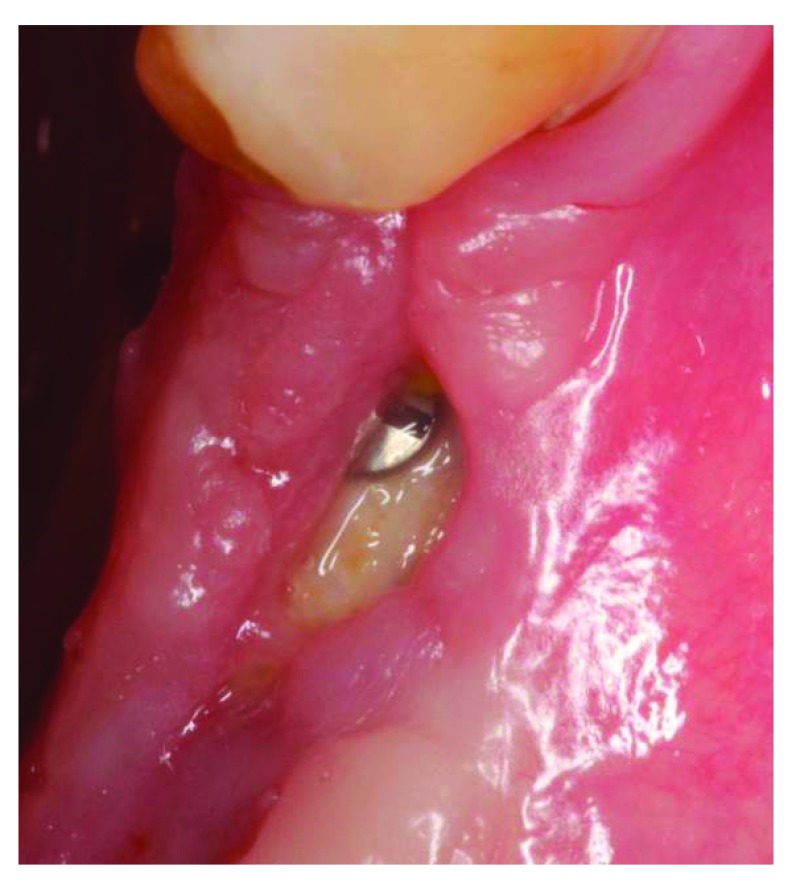
4 weeks follow-up, membrane exposure can be observed.

**Figure 11.**
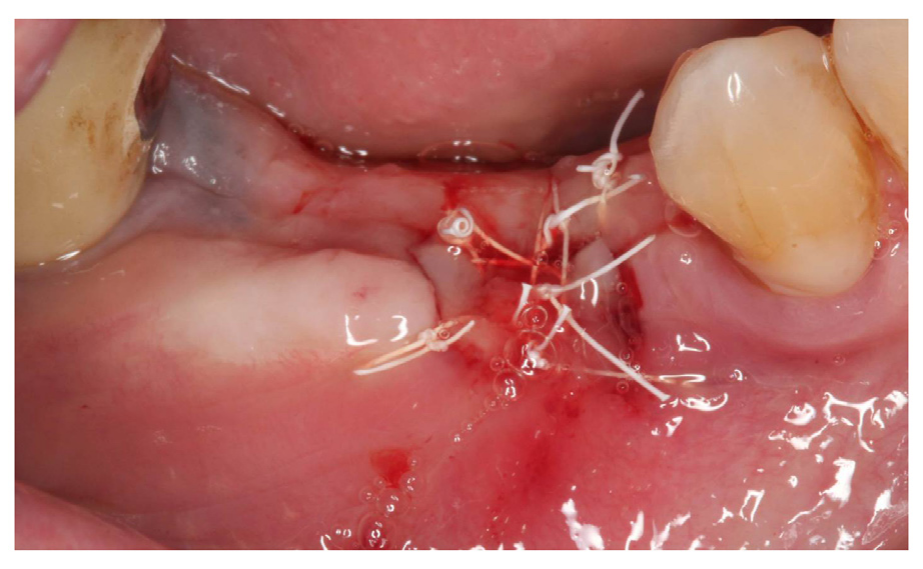
Membrane covering surgery.

At the 4-week follow up point, clinical examination revealed that there was post-operative exposure of the membrane. The size of the exposure was approximately 4 × 8 mm ( Figure 9 and Figure 10). The sutures were removed and it was decided to manage the exposure surgically by making two small vertical incisions and positioning the tissue coronally to cover the membrane. Non-resorbable sutures 4-0 (Cytoplast™ PTFE) were used ( Figure 11). Patient was instructed to use chlorhexidine mouthwash and weekly recall to monitor the surgical site.

**Figure 12.**
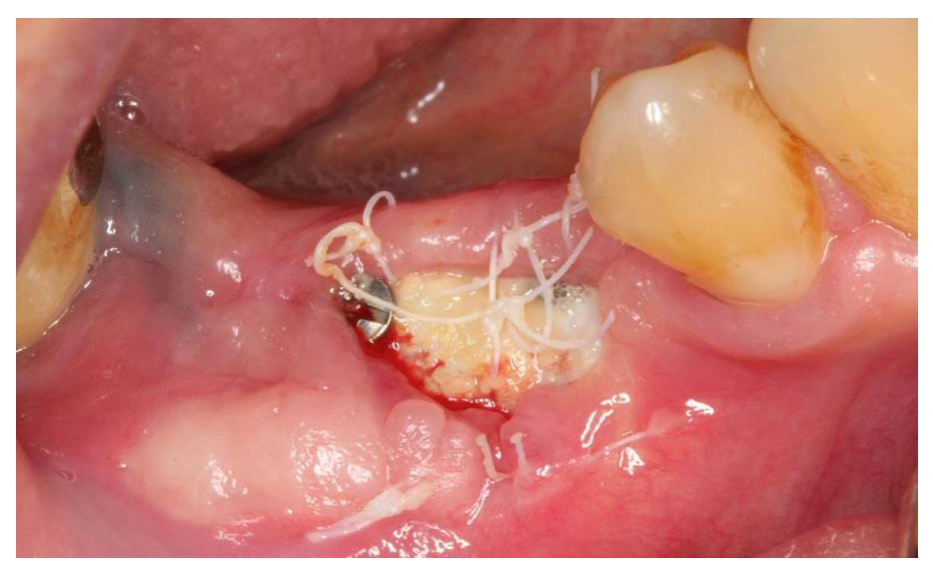
The patient at 1 week after membrane covering surgery.

At the 5-week follow up point, 1 week after our surgical attempt to cover the membrane exposure, the size of exposure had increased ( Figure 12). Sutures were removed and the outer surface of the membrane was cleaned with cotton swap dipped in chlorohexidine. The patient was instructed to use cotton swab dipped in chlorohexidine to clean the exposed membrane.

**Figure 13.**
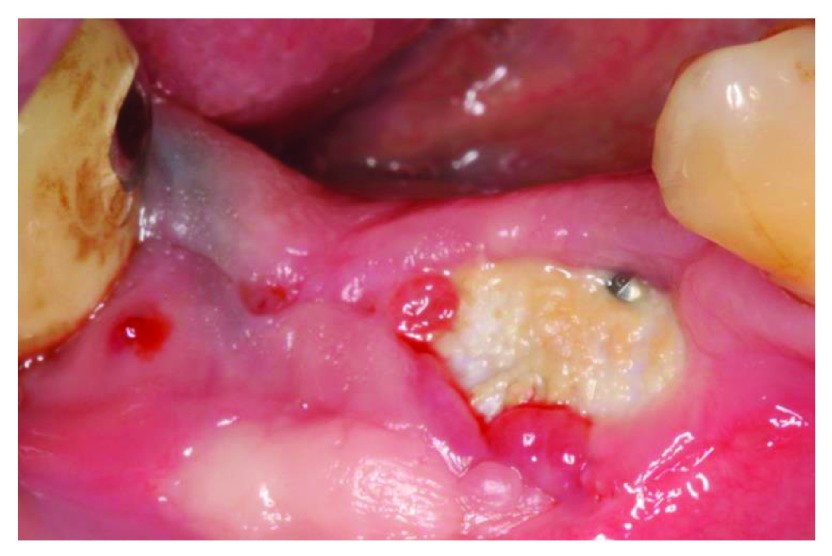
The patient at 2 weeks after membrane covering surgery.

**Figure 14.**
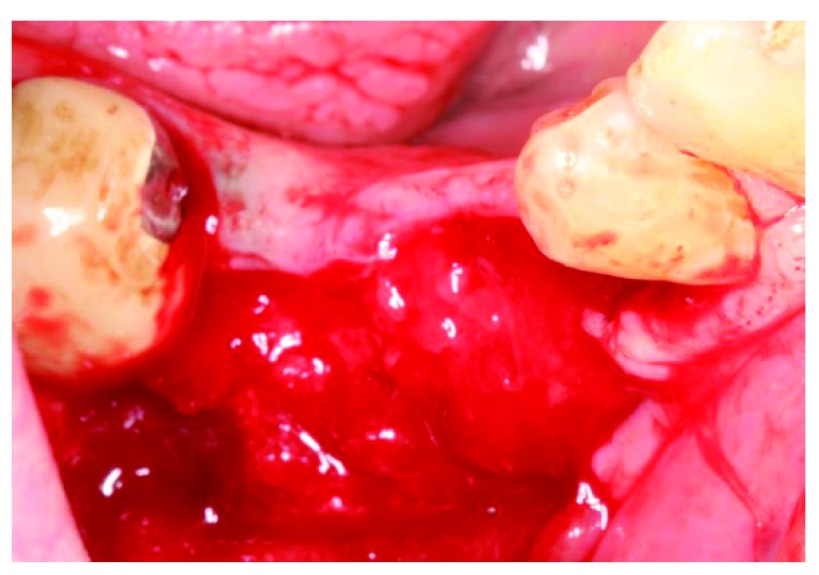
The underlying tissue after membrane removal. No bone graft remnants were observed in the surgical site.

**Figure 15.**
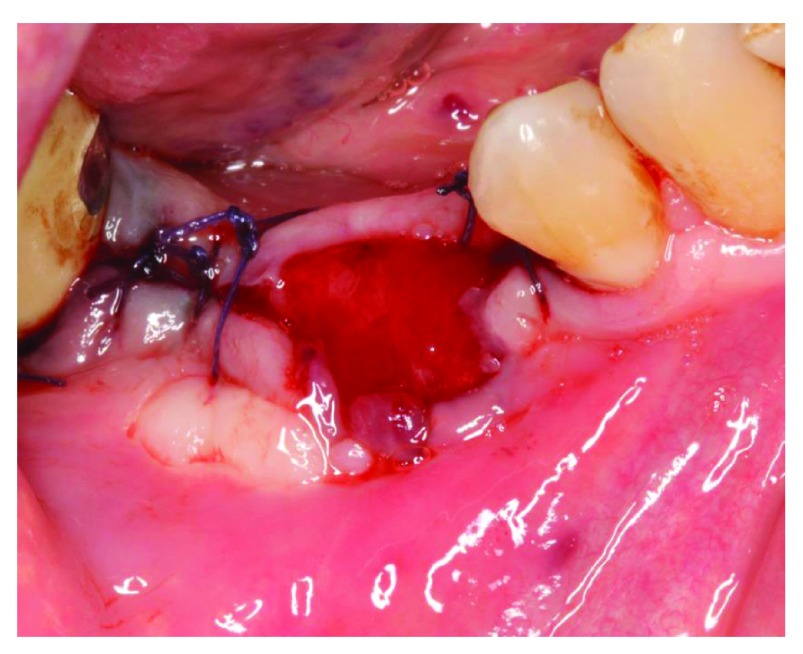
Suturing after membrane removal.

At the 6-week follow-up point, 2 weeks after the membrane covering procedure, intraoral examination revealed pus discharge between the membrane and the tissue ( Figure 13). The membrane had to be removed owing to the infection. An incision was made to split them membrane from the tissue then the membrane was removed ( Figure 14). After removing the membrane, the underlying tissue had a red, jelly-like appearance, with no bone -graft remnants observed in the surgical site. The flap was sutured using resorbable sutures ( Figure 15). The patient was instructed to take 1 g Augmentin twice daily for 1 week and to use 0.12% chlorhexidine mouth wash twice a day for 2 weeks. 

**Figure 16.**
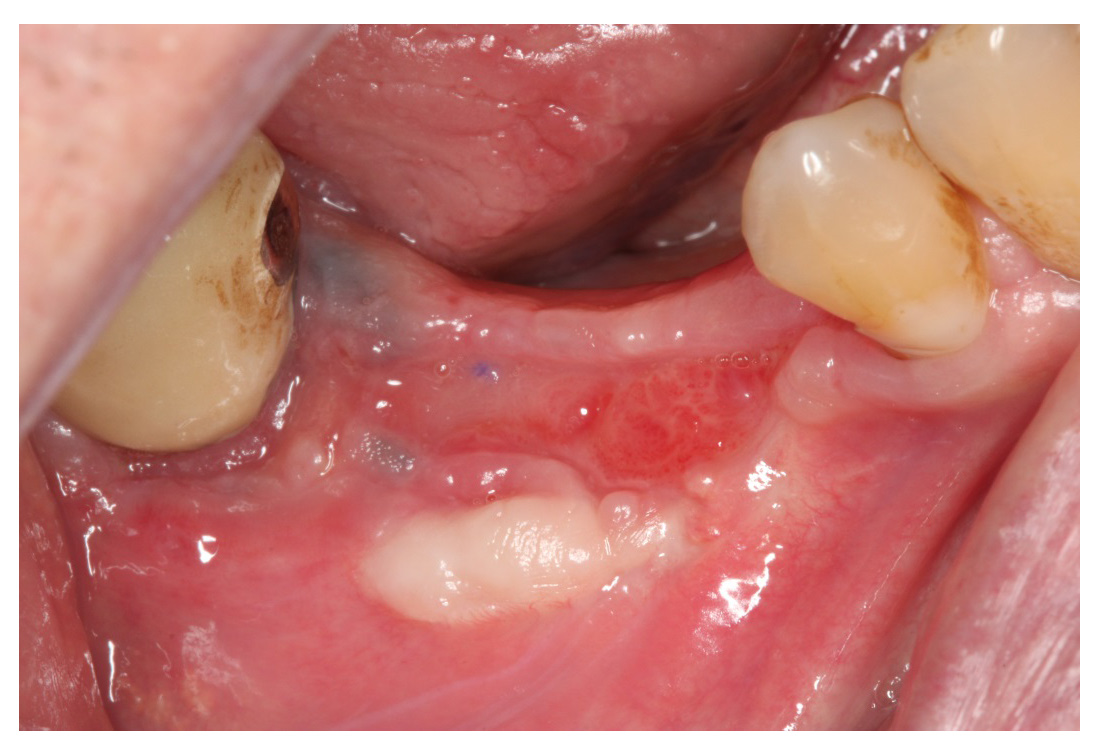
The patient at 2 weeks after membrane removal.

At 8 weeks after the horizontal ridge augmentation, the surgical site was healing well with no sign of infection ( Figure 16).

### Implant placement

**Figure 17.**
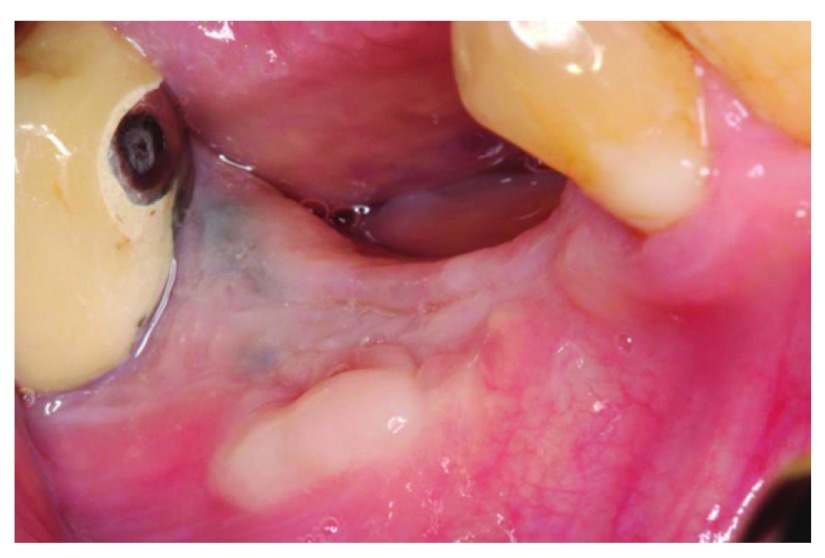
The patient at 5 months after guided bone regeneration, the day of implants placement.

At 5 months after the guided bone regeneration (GBR) procedure, a cone-beam CT scan was performed to evaluate the bone width. The bone width was 9 mm, meaning that the bone width gain after ridge augmentation was 6 mm. two implants were successfully placed at site #46 and #45 ( Figure 17 and Figure 19).

**Figure 18.**
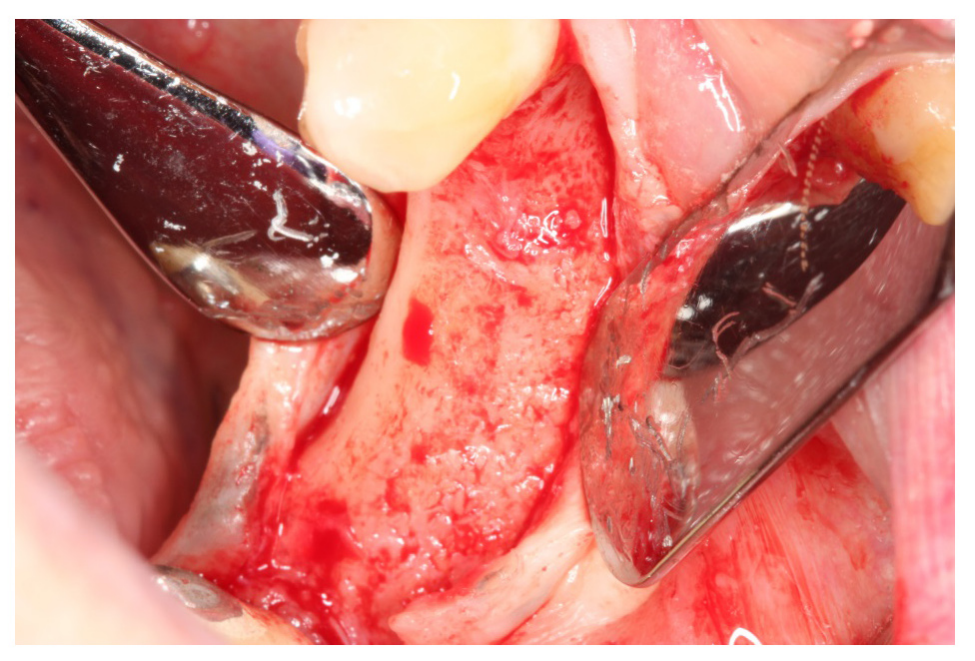
Full-thickness flap reflection. The bone width gain after guided bone regeneration can be noticed.

**Figure 19.**
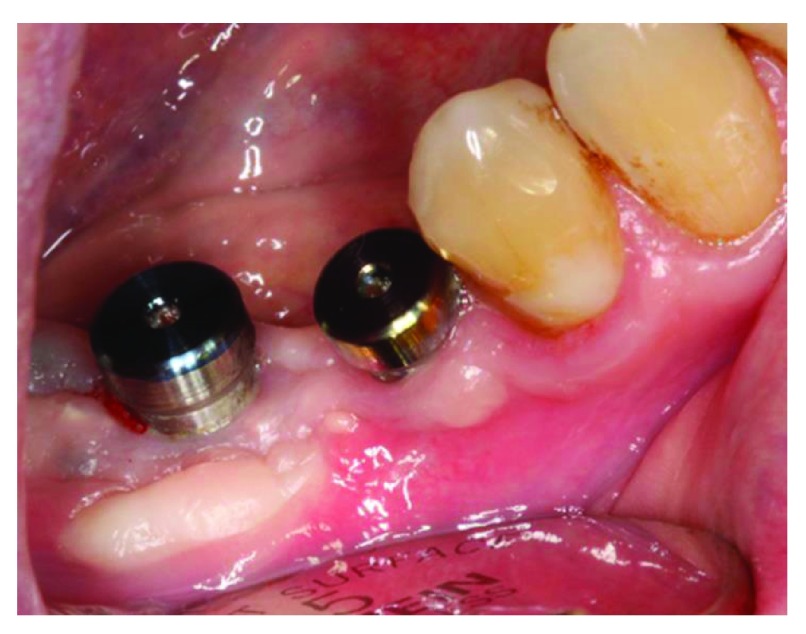
Implant placement.

## Discussion

The purpose of presenting this clinical case was brought out that horizontal ridge augmentation using a combination of titanium-reinforced non-resorbable PTFE membrane and FDBA resulted in the successful implants placement at the sites #46 and #45 despite the membrane exposure that occurred at 4 weeks following horizontal ridge augmentation, the infection that had occurred after a further 2 weeks.

There are four types of non-resorbable membranes, dense PTFE, expanded PTFE), titanium mesh, and titanium-reinforced polytetrafluoroethylene. In this case, titanium-reinforced polytetrafluoroethylene was used. As with other types of non-resorbable membranes, the most common complication is post-operative exposure ^6^. The rate of membrane exposure following guided bone regeneration is 31%, GBR failure due to membrane exposure have been reported ^7^. Membrane exposure permits a communication between the oral environment and the newly forming tissues, increasing the potential for infection and decreasing the likelihood of regeneration ^8^. In this case, we attempted to manage the exposure surgically by advancing the tissue coronally to cover the exposed membrane, with the aim of preventing the communication between the oral environment and the newly forming tissue. Our second option to manage the exposure was frequent patient follow-up and maintaining good oral hygiene during the healing period.

 To best of our knowledge, no experimental or clinical studies have been conducted to study the proper management of post-operative membrane exposure. In addition to that, there is a disagreement about the impact of membrane exposure on bone regeneration. According to Rita A *et al.* ^8^ membrane exposure remarkably reduces bone fill, and less bone regeneration around immediate implants sites is observed when exposed membranes are compared to non-exposed membrane sites whereas in a retrospective study where 237 sites treated with guided tissue regeneration were examined, Shanaman ^9^ found that exposure of membrane had no negative impact on bone regeneration if the patient maintained adequate postoperative oral hygiene. The size of exposure increased after our surgical attempt to advance the tissue coronally, however, 1 week after the surgery, no sign of infection was observed which allowed us to keep the membrane in its place. The size of the exposure had increased due to flap sloughing over the membrane owing to inadequate blood supply, which could be explained by the size of the flap and narrow flap base. We did not widen the base of the flap due to the risk of compromising the unexposed membrane. The patient was recalled weekly to check the site. At 2 weeks after the membrane covering surgery, pus discharge between the membrane and the surrounding tissue was noticed. At that time, removal of the membrane was required to avoid the spread of infection to the newly forming tissue. The membrane was removed at 6 weeks after the ridge augmentation procedure. After removing the membrane; the underlying tissue had a red - jelly like appearance with no bone graft remnants observed in the surgical site.

At 5 months after GBR, the bone width was 9 mm, and eventually, the bone gain was 6 mm, which was assessed with a cone-beam CT scan. Subsequently, two implants were successfully placed at site #46 and #45.

 The present case study shows the unpredictability of managing postoperative membrane exposure surgically. It also, shows that the ridge augmentation was successful after removing the non-resorbable membrane at 6 weeks after the ridge augmentation procedure. Further studies are required regarding the proper management of post-operative membrane exposure.

## Data availability

All data underlying the results are available as part of the article and no additional source data are required.

## Consent

Written informed consent for publication of their clinical details and images was obtained from the patient.
