# Supplementary material for: Case Report: Managing the postoperative exposure of a non-resorbable membrane surgically
Source: F1000Res. 2018 May 31;7:685. [Version 1] doi: 10.12688/f1000research.14939.1 (PMC6085604; doi:10.12688/f1000research.14939.1)
Supplement: Peer review history

# Peer review: Referee response for version 1

**DOI:** 10.5256/f1000research.16264.r36238

**Published:** 2018-08-10

**Authors:** Zachary Evans

**Affiliations:** 1 Department of Stomatology, Medical University of South Carolina, Charleston, SC, USA

This was an interesting report of a common clinical occurrence relating to membrane exposure following oral guided bone regeneration procedures.  Some existing evidence suggests that exposure is more common with non-resorbable membranes as seen in this report.  This is a very well documented progression showing and discussing the progression of exposure, infection, surgical intervention, and resolution.  The report mentions several times the observation of the “red jelly like” substance.  This is clearly osteoid that has been formed and not lost due to the infection.  Some research is available discussing the speed of formation (1-3 weeks post grafting, but not much is available discussing the formation and stability in the setting of membrane exposure or infection.  Therefore, I would recommend this manuscript be considered for indexing with the addition of a brief review or mention of osteoid formation timeline in the discussion.

I have read this submission. I believe that I have an appropriate level of expertise to confirm that it is of an acceptable scientific standard, however I have significant reservations, as outlined above.

# Peer review: Referee response for version 1

**DOI:** 10.5256/f1000research.16264.r36237

**Published:** 2018-08-09

**Authors:** Khalid Almas

**Affiliations:** 1 Department of Preventive Dental Sciences, Imam Abdulrahman Bin Faisal University, Dammam, Saudi Arabia

This manuscript is contemporary as far as the scientific writing and the clinical procedures are concerned. The case report is an effort to highlight the complication of ridge augmentation surgery, which is not uncommon. The case has been managed successfully.

 All the parameters are at par with current routine clinical practice.

I have read this submission. I believe that I have an appropriate level of expertise to confirm that it is of an acceptable scientific standard.

# Peer review: Referee response for version 1

**DOI:** 10.5256/f1000research.16264.r34962

**Published:** 2018-07-16

**Authors:** Gerald T. Grant

**Affiliations:** 1 Department of Oral Health and Rehabilitation, University of Louisville School of Dentistry, Louisville, KY, USA

As a case study it does exemplify some of the issues that are seen with bone augmentation and working with membranes.  The discussion is appropriate and the observations seem to be accurate. 

 Both the photos and the radiographs contribute to the paper as they show the issues as they were addressed.

I have read this submission. I believe that I have an appropriate level of expertise to confirm that it is of an acceptable scientific standard.
